# A novel fibroblast growth factor receptor 1 inhibitor protects against cartilage degradation in a murine model of osteoarthritis

**DOI:** 10.1038/srep24042

**Published:** 2016-04-04

**Authors:** Wei Xu, Yangli Xie, Quan Wang, Xiaofeng Wang, Fengtao Luo, Siru Zhou, Zuqiang Wang, Junlan Huang, Qiaoyan Tan, Min Jin, Huabing Qi, Junzhou Tang, Liang Chen, Xiaolan Du, Chengguang Zhao, Guang Liang, Lin Chen

**Affiliations:** 1 Department of Rehabilitation Medicine, Center of Bone Metabolism and Repair, State Key Laboratory of Trauma, Burns and Combined Injury, Trauma Center, Research Institute of Surgery, Daping Hospital, Third Military Medical University, Chongqing 400042, China; 2 Institute of Biological and Natural Medicine, School of Pharmaceutical Science, Wenzhou Medical University, Wenzhou 325035, China

## Abstract

The attenuated degradation of articular cartilage by cartilage-specific deletion of fibroblast growth factor receptor 1 (FGFR1) in adult mice suggests that FGFR1 is a potential target for treating osteoarthritis (OA). The goal of the current study was to investigate the effect of a novel non-ATP-competitive FGFR1 inhibitor, G141, on the catabolic events in human articular chondrocytes and cartilage explants and on the progression of cartilage degradation in a murine model of OA. G141 was screened and identified via cell-free kinase-inhibition assay. In the *in vitro* study, G141 decreased the mRNA levels of catabolic markers ADAMTS-5 and MMP-13, the phosphorylation of Erk1/2, JNK and p38 MAPK, and the protein level of MMP-13 in human articular chondrocytes. In the *ex vivo* study, proteoglycan loss was markedly reduced in G141 treated human cartilage explants. For the *in vivo* study, intra-articular injection of G141 attenuated the surgical destabilization of the medial meniscus (DMM) induced cartilage destruction and chondrocyte hypertrophy and apoptosis in mice. Our data suggest that pharmacologically antagonize FGFR1 using G141 protects articular cartilage from osteoarthritic changes, and intra-articular injection of G141 is potentially an effective therapy to alleviate OA progression.

OA is a serious public health problem worldwide that can lead to significant disability and severe joint pains, severely influencing the life quality of patients. Thus far, there is few biological and pharmacological treatments available to prevent the structural damage caused by OA. Ultimately, most of the OA patients have to undergo joint replacement surgeries when their joints are functionally failed^1^. Therefore, finding effective therapies to delay the progression of cartilage destruction in patients with OA is a critical medical priority.

In recent years, a variety of molecules and signaling pathways in the articular chondrocytes, such as mTOR, IHH and Wnt/β-catenin signaling, have been found to be involved in cartilage homeostasis and OA development^2^,^3^,^4^. Fibroblast growth factors (FGFs) and their receptors (FGFRs) regulate the maintenance of cartilage and therefore, play vital roles in cartilage homeostasis and OA development^5^. FGFR1 and FGFR3 are the predominant receptors expressed in human chondrocytes^6^. Valverde-Franco and colleagues demonstrated the protective effect of FGFR3 on mouse joint cartilage by revealing spontaneous OA in Fgfr3-deletion mice with accelerated cartilage degradation associated with increased levels of extracellular matrix degrading enzymes including MMP-13^7^. With regard to FGFR1, our group demonstrated that FGFR1 mediates catabolic activities in cartilage and is associated with an increase of MMP-13 expression and downregulation of proteoglycan synthesis, and conditional knockout of Fgfr1 in cartilage delays the progression of cartilage degradation in aging and surgically induced mouse OA models^8^. In human articular chondrocytes, FGF-2 selectively activates FGFR1 to exert catabolic effects via up-regulation of MMP-13, inhibition of proteoglycan synthesis and ECM accumulation^9^. These evidences reveal that FGFR1 is a potential therapy target for treating OA and pharmacological FGFR1 antagonists may prevent cartilage degradation and/or improve cartilage homeostasis.

At present, several small molecules, such as PD173074, SU5402 and PD166866, have been used as FGFR tyrosine kinase inhibitors^10^. These inhibitors were designed based on their competitive inhibition of the ATP-binding domain of FGFR1. However, the ATP-binding sites are highly conservative among majority of the tyrosine kinases, these small molecules exhibit poor selectivity profile and their drug potency is easily affected by the high intracellular ATP concentration. The non-ATP-competitive inhibitors, which bind to the non-ATP binding site, possess the superior selectivity^11^. We have identified several non-ATP-competitive FGFR1 inhibitors via kinase inhibition assay of a chemical bank that containing 156 bisaryl-1, 4-dien-3-one compounds, and these inhibitors specifically target FGFR1 with weak effect on other tyrosine kinases^10^,^11^.

In this study, we analyzed the impact of a novel non-ATP-competitive FGFR1 inhibitor, G141, on FGF-2 or IL-1β–induced catabolic events in human articular chondrocytes and cartilage explants. Furthermore, we performed intra-articular injection of G141 into mouse knee joints in a DMM model of OA, to examine whether G141 inhibits cartilage degradation during OA. Our observations suggest that G141 reduces the catabolic events in FGF-2 or IL-1β treated human articular chondrocytes and human cartilage explants, and intra-articular injection of G141 protects articular cartilage from degradation after DMM in mice.

## Results

### G141 inhibits the activity of FGFR1 selectively in an ATP independent manner

**Figure 1:**
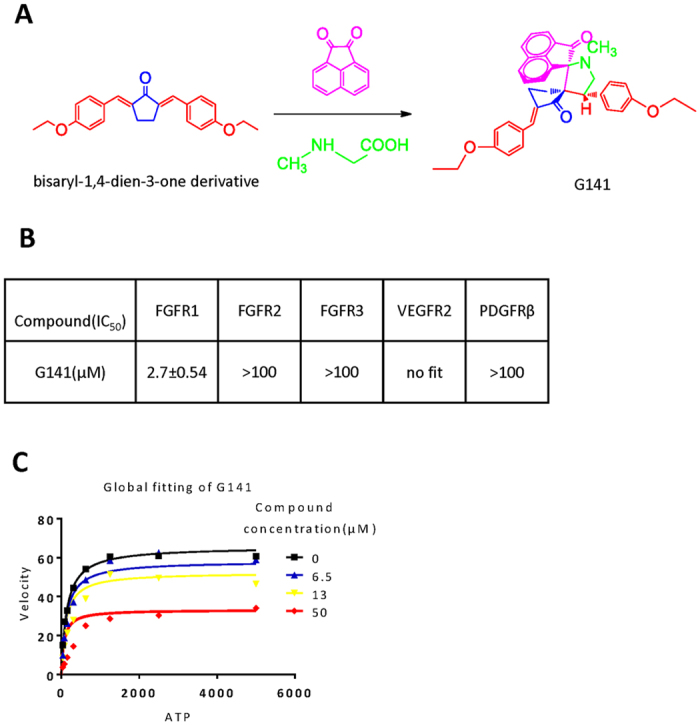
G141 inhibits FGFR1 activity in a non-ATP competitive manner. (**A**) Molecular scheme of bisaryl-1, 4-dien-3-one derivative and G141. (**B**) G141 selectively inhibited FGFR1. G141 were tested with caliper mobility shift assay for RTKs inhibition, and the IC_50_ values were calculated using conversion rates. The data were shown as a mean of 3 independent tests. (**C**) G141 inhibited FGFR1 through a mechanism that was independent of the concentrations of ATP. Selective ATP-competitive kinase assay of G141 with FGFR1 was carried out through caliper mobility shift assay. The conversion data were fitted with Graphpad for global fitting.

Previously, we designed a library of bisaryl-1, 4-dien-3-one compounds to screen and identify FGFR1 inhibitors^11^. In this study, G141 was found to have high affinity for FGFR1 (IC_50_: 2.7 ± 0.54 μM) (Fig. 1A). To test the specificity of G141, we further measured the inhibitory effect of G141 on other receptor tyrosine kinases (RTKs), including VEGFR2, PDGFRβ, FGFR2 and FGFR3. As displayed in Fig. 1B, G141 showed a much lower activity against these RTKs compared to that of FGFR1. These data demonstrated that G141 selectively inhibited the activity of FGFR1.

Subsequently, we used caliper mobility shift assay to study the competitive relationship between ATP and G141. As shown in Fig. 1C, the increased concentration of ATP did not affect the rate of FGFR1 substrate phosphorylation at the various concentrations of G141. In other words, the inhibition of FGFR1 kinase activity by G141 did not depend on the concentrations of ATP. Thus, G141 suppressed FGFR1 in an ATP-independent manner.

### G141 inhibits the expressions of catabolic markers in FGF-2 treated human articular chondrocytes

**Figure 2:**
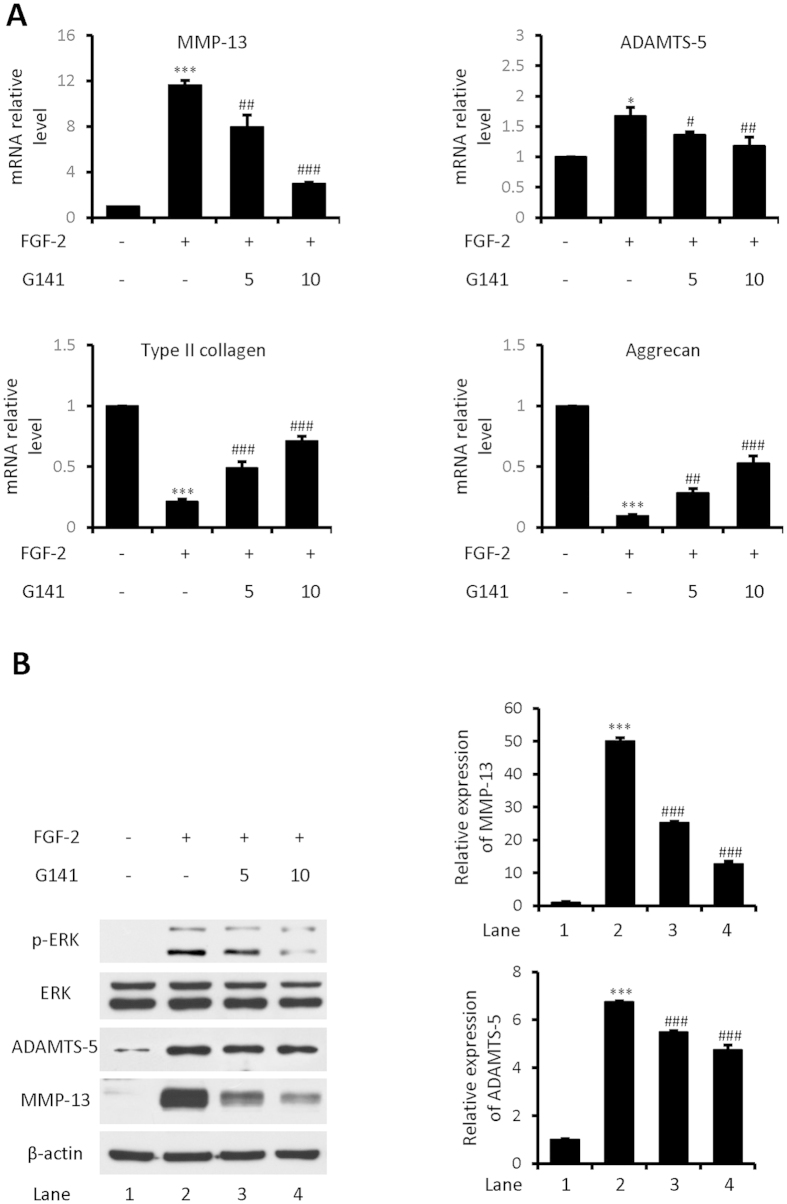
Effects of G141 on the mRNA levels of MMP-13, ADAMTS-5, type II collagen and aggrecan and on MMP-13, ADAMTS-5 and p-ERK1/2 protein levels in FGF-2 treated human articular chondrocytes. Human articular chondrocytes were treated with G141 (5 μM, 10 μM) for 1 hour, followed by treatment with FGF-2 (20 ng/ml) for 24 hours. (**A**) Total RNA were isolated, and levels of mRNAs for the catabolic markers (MMP-13 and ADAMTS-5) and articular chondrocyte markers (type II collagen and aggrecan) were detected by Real-time qPCR (Values are the mean ± SEM. n = 3, X ^*^p < 0.05 versus controls, ^***^p < 0.001 versus controls, ^#^p < 0.05 versus FGF-2 alone, ^##^p < 0.01 versus FGF-2 alone, ^###^p < 0.001 versus FGF-2 alone). (**B**) Cell lysates were analyzed by Western blotting using antibodies specific for MMP-13, ADAMTS-5, phosphorylated and total ERK1/2. The signal intensities of MMP-13 and ADAMTS-5 were quantified using software ImageJ (version 1.47). (n = 3, ^***^p < 0.001 versus controls, ^###^p < 0.001 versus FGF-2 alone).

As FGF-2 induced catabolic and anti-anabolic activities in human articular chondrocytes mainly via FGFR1, we first examined whether G141 could abolish the catabolic and anti-anabolic effect of FGF-2 on human articular chondrocytes. Primary human articular chondrocytes in monolayer were pre-incubated with G141 (5 μM, 10 μM) followed by stimulation with FGF-2 (20 ng/mL) for 24 hours. Real-time qPCR was performed to examine the effects of G141 on markers of extracellular matrix synthesis and breakdown. The mRNA levels of ADAMTS-5 and MMP-13 were significantly increased after FGF-2 treatment. G141 treatment resulted in a marked reduction in these two catabolic markers in human articular chondrocytes treated with FGF-2. In contrast, after FGF-2 treatment, the mRNA levels of cartilage markers aggrecan and type II collagen were markedly reduced in human articular chondrocytes, which were partially recovered by G141 treatment (Fig. 2A).

Since FGFR1-Ras/PKCδ–Raf–MEK1/2–ERK1/2 signaling pathway plays a major role in the FGF-2–mediated stimulation of extracellular matrix degrading enzymes (ADAMTS-5 and MMP-13) in human articular chondrocytes^12^, we examined whether treatment with G141 could affect the signaling activity of ERK1/2 and protein levels of ADAMTS-5 and MMP-13 in human articular chondrocytes treated with FGF-2. We analyzed samples from total cell lysates of human articular chondrocytes cultured in the absence or presence of FGF-2 and G141, by immunoblotting with antibodies specific for phosphorylated and total ERK1/2, ADAMTS-5 and MMP-13. Following stimulation of FGF-2, activation of ERK1/2 MAPK signaling pathway was evident, and the protein levels of ADAMTS-5 and MMP-13 were largely induced. In the presence of G141, the degree of increase in the protein levels of phosphorylated ERK1/2, ADAMTS-5 and MMP-13 were attenuated in FGF-2–treated human articular chondrocytes (Fig. 2B).

**Figure 3:**
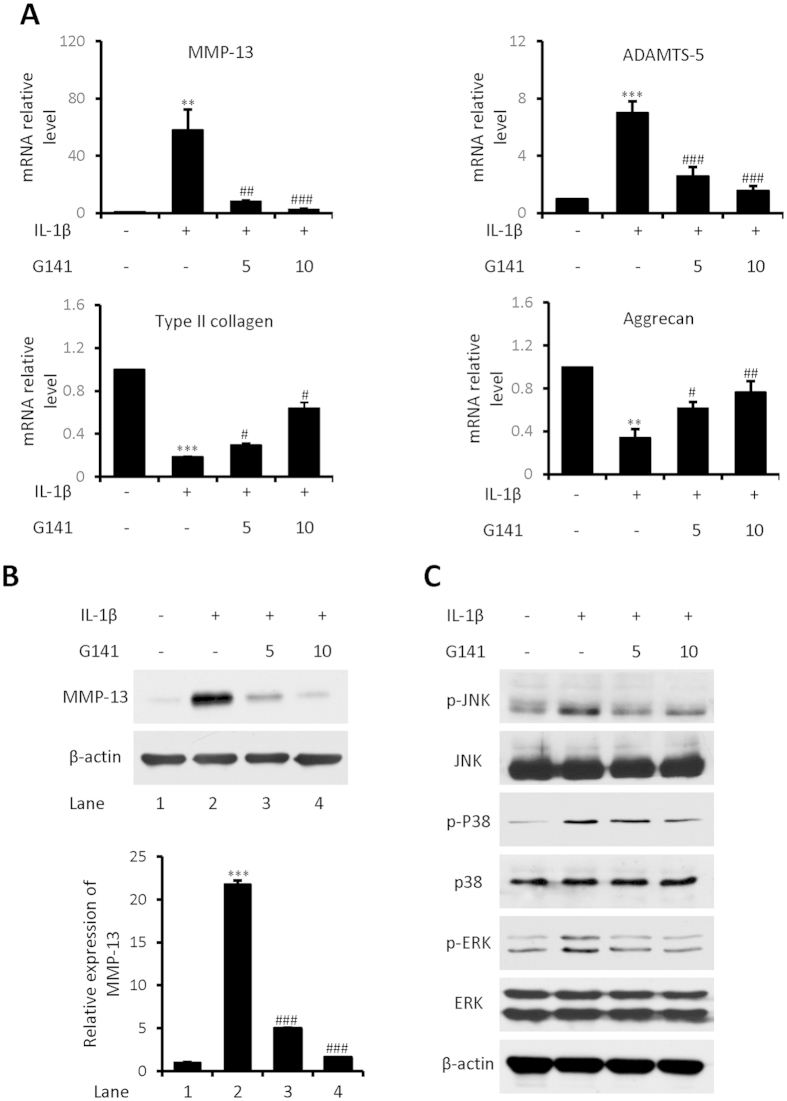
Effects of G141 on mRNA levels of MMP-13, ADAMTS-5, type II collagen and aggrecan and on MMP-13, p-JNK, p-ERK1/2 and p-p38 protein levels in IL-1β treated human articular chondrocytes. Human articular chondrocytes were treated with G141 (5 μM, 10 μM) for 1 hour, followed by treatment with IL-1β (20 ng/ml) for 24 hours. (**A**) Total RNA were isolated, and levels of mRNA of the catabolic markers (MMP-13 and ADAMTS-5) and articular chondrocyte markers (type II collagen and aggrecan) were detected by Real-time qPCR. (Values are the mean ± SEM. n = 3, ^**^p < 0.01 versus controls, ^***^p < 0.001 versus controls, ^#^p < 0.05 versus IL-1β alone, ^##^p < 0.01 versus IL-1β alone, ^###^p < 0.001 versus IL-1β alone). (**B**) Cell lysates were analyzed by Western blotting using antibodies specific for MMP-13, phosphorylated and total JNK, ERK1/2 and p38. The signal intensities of MMP-13 were quantitatively analyzed (Values are the mean ± SEM, n = 3, ^***^p < 0.001 versus controls, ^###^p < 0.001 versus IL-1β alone).

IL-1β has been shown to play a prominent role in cartilage degradation by inhibiting ECM synthesis and promoting cartilage breakdown. We determined whether G141 treatment could affect the catabolic events in human articular chondrocytes initiated by IL-1β. G141 treatment resulted in a remarkable reduction in the IL-1β up-regulated mRNA levels of ADAMTS-5 and MMP-13, and a marked increase in the IL-1β down-regulated mRNA levels of aggrecan and collagen type II (Fig. 3A). Western blotting result showed that G141 attenuated the degree of increase in the protein levels of MMP-13 and phosphorylated JNK, ERK1/2 and p38 MAPK resulting from IL-1β treatment in chondrocytes (Fig. 3B,C).

### G141 reduces the loss of proteoglycan in cultured human articular cartilage explants

**Figure 4:**
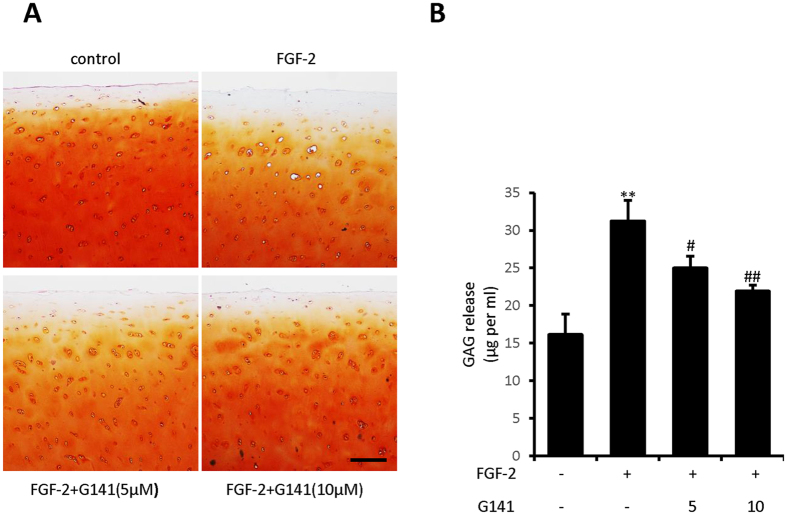
Effects of G141 on proteoglycan loss in adult human articular cartilage. Full thickness cartilage explants from human femur head were cultured in the absence or presence of FGF-2 (50 ng/ml) and in the presence or absence of G141 (5 μM, 10 μM) for 14 days. (**A**) 5 μm Paraffin sections were stained with Safranin O-fast green to identify the cartilage proteoglycan loss. Scale bar, 200 μm. (**B**) Culture medium were collected and the amount of GAG released into the medium were quantified by DMMB assay. GAG released into the medium was normalized as mass of GAG per milliliter (ml) of culture medium (Values are the mean ± SEM of 3 independent trials per treatment group, ^*^p < 0.05 versus controls, ^#^p < 0.05 versus FGF-2 alone, ^##^p < 0.01 versus FGF-2 alone).

To examine the effect of G141 on proteoglycan loss, we cultured human femoral head cartilage samples in the absence or presence of FGF-2 and G141 for 14 days. Safranin-O-fast green staining showed that FGF-2 (50 ng/ml) significantly induced proteoglycan depletion, which was partially rescued by G141 treatment (Fig. 4A). Culture medium was also collected to analyze the release of GAG using DMMB assay. G141 treatment markedly decreased the release of GAG into the culture medium from FGF-2–treated human femoral head cartilage samples (Fig. 4B).

### G141 delays articular cartilage degradation in a mouse model of DMM

**Figure 5:**
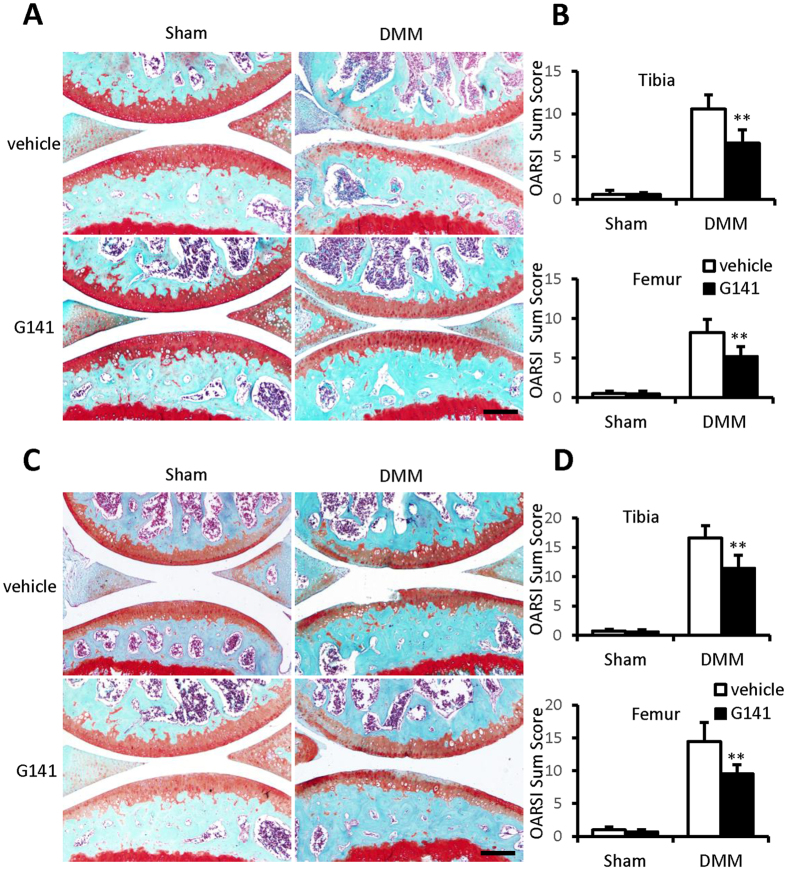
Effects of G141 on cartilage degradation in mouse articular cartilage at 4 and 8 weeks after surgical destabilization of the medial meniscus (DMM). 10-week-old male C57BL/6 mice were subjected to sham operation or DMM surgery followed by intra-articular injection of 10 μM G141 or PBS twice a week for 4 and 8 weeks, immediately after DMM surgery. (**A**) Knee joints were harvested at 4 weeks post-surgery and analyzed histologically by Safranin O-fast green staining. Representative images were shown. Scale bar, 200 μm. (**B**) The joints were scored histologically using the OARSI scoring system at 4 weeks post-surgery. (**C**) Knee joints were harvested at 8 weeks post-surgery and analyzed histologically by Safranin O-fast green staining. Representative images were shown. Scale bar, 200 μm. (**D**) The joints were scored histologically using the OARSI scoring system at 8 weeks post-surgery. Results are the mean ± SEM from 6 mice per group. ^**^P < 0.01 versus vehicle-treated group.

Surgical destabilization of the medial meniscus (DMM) in mice is a well-established model of OA, which is characterized with articular cartilage degradation including abrasion of the articular surfaces, and up-regulated levels of pro-inflammatory cytokines (e.g. IL-1) and catabolic molecules (e.g. ADAMTS5, MMP13)^5^,^15^. This mouse OA model is commonly used to screen biological and pharmacological agents for OA treatment^13^,^14^. To examine the effect of G141 on the development of OA induced by injury, we performed DMM surgery in the right knee joints of 10-week-old male C57 mice. After DMM surgery, the mice received twice weekly intra-articular injection of 10 μM G141 in PBS or PBS alone for 2, 4 and 8 weeks. Total RNA and protein from mouse knee joints 2 weeks following DMM or sham surgery were extracted. The mRNA levels of IL-1β and FGF-2 were significantly increased in joints after DMM surgery compared with that in sham operated control joints. Intra-articular injection of G141 resulted in a marked reduction of the mRNA levels of IL-1β and FGF-2 in joints after DMM surgery (see Supplementary Fig. S1A). The level of phosphorylated FGFR1 was increased in joints after DMM surgery, which was attenuated by G141 treatment (see Supplementary Fig. S1B). These data demonstrated that G141 inhibited the activity of FGFR1 and IL-1β expression in mouse knee joints after DMM surgery. Safranin O–fast green staining results demonstrated significant reduction in proteoglycan loss, cartilage destruction and loss of articular chondrocyte cellularity in mice treated with G141 at 4 and 8 weeks after DMM surgery compared to the DMM mice treated with vehicle (Fig. 5A,C).

OARSI histologic scoring system was applied to quantitatively analyze the cartilage degradation after DMM surgery. The summed OARSI score demonstrated that G141-treated mice had a significantly lower score than the vehicle-treated mice at 4 and 8 weeks following DMM surgery. The summed OARSI score in the G141-treated mice at 8 weeks was increased compared to the score at 4 weeks. These findings suggested that intra-articular injection of G141 did not completely prevent but delayed cartilage degradation in mouse knee joint after DMM surgery (Fig. 5B,D).

### G141 attenuates chondrocyte hypertrophy in knee joints of DMM mice

**Figure 6:**
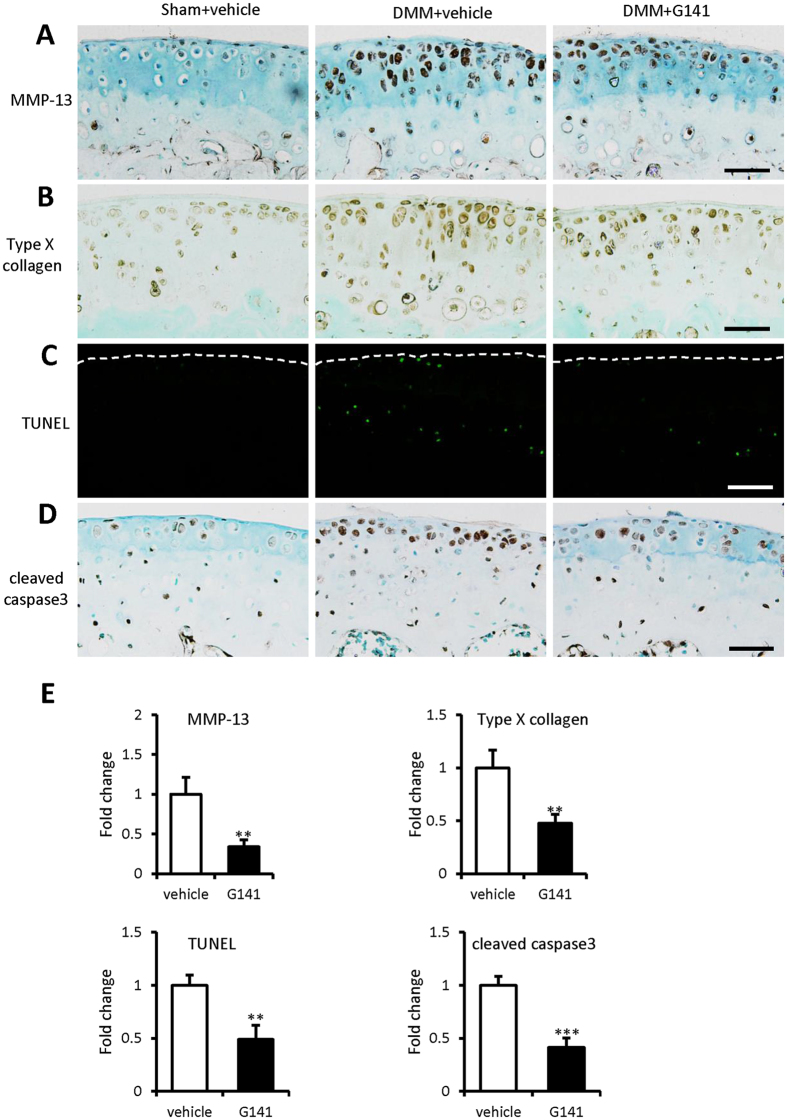
Effects of G141 on chondrocyte hypertrophy and apoptosis in mouse articular cartilage at 8 weeks after DMM. The knee joints were immunohistochemically stained for MMP-13 (**A**), type X collagen (**B**) and cleaved caspase-3 (**D**) at 8 weeks after DMM with intra-articular injection of G141 or vehicle. (**C**) TUNEL assay was performed on knee joints to measure chondrocyte apoptosis at 8 weeks after DMM with intra-articular injection of G141 or vehicle. Scale bar, 50 μm. (**E**) Fold changes of MMP-13, type X collagen, TUNEL and cleaved caspase-3 positive cells in the articular cartilage were calculated. The percentage of positive cells in vehicle-treated group were defined as 1. Values are the mean ± SEM of 3 independent trials per treatment group. ^**^P < 0.01 versus vehicle-treated group,^***^P < 0.001 versus vehicle-treated group.

To explore the mechanisms underlying the delayed progression of cartilage degradation in G141-treated mice with DMM, we performed immunohistochemical staining to examine the expressions of type X collagen and MMP-13, marker gene for hypertrophic articular chondrocytes. The results showed that G141 treatment significantly decreased the number of MMP-13 (Fig. 6A,E) and type X collagen (Fig. 6B,E) positive cells by 66% and 52%, respectively, in the knee joints 8 weeks after DMM surgery compared to vehicle treatment. These results suggested that local intra-articular injection of G141 reduced articular cartilage damage, at least in part, through inhibition the hypertrophy process of articular chondrocytes.

### G141 decreases chondrocyte apoptosis in knee joints of DMM mice

Since it has been reported that chondrocyte apoptosis is strongly related to OA development^16^,^17^, apoptosis was examined using TUNEL assay and immunohistochemical staining of cleaved caspase-3, a marker to measure apoptosis. TUNEL staining showed that G141 treatment largely decreased chondrocyte apoptosis by 51% in the knee joints 8 weeks after DMM surgery compared to vehicle treatment (Fig. 6C,E). The number of cleaved caspase-3-positive cells in the articular cartilage was markedly decreased by 59% in the G141 group compared to the vehicle group at 8 week post DMM surgery (Fig. 6D,E). Taken together, local intra-articular injection of G141 protected chondrocytes from apoptosis in mice with DMM surgery.

## Discussion

OA is a degenerative disease characterized with cartilage degradation, synovial inflammation and dysregulated subchondral bone remodeling. The mechanisms underlying OA is not well understood, and there is currently few effective treatment to prevent the development of OA, joint replacement is usually carried out in patients with severe OA. It is urgent to find effective therapies to prevent or slow down the progression of OA.

Decades of studies have demonstrated that fibroblast growth factors (FGFs) and their receptors (FGFRs) regulate the development and maintenance of cartilage, and therefore play vital roles in cartilage homeostasis and OA development^5^. We previously demonstrated that conditional deletion of Fgfr1 in mature mouse articular chondrocytes delays the progression of cartilage degradation^8^. Here, we revealed that pharmacologically antagonize FGFR1 using G141, a novel non-ATP-competitive inhibitor, can attenuate the development of OA.

Four members of the FGF family, FGF-2, FGF-8, FGF-9, and FGF-18, have been reported as key factors regulating cartilage degradation and homeostasis. Recently, studies have begun to explore the potential therapeutic effect of these biological agents. FGF-18 is a well-established anabolic growth factor that induces cartilage ECM formation^18^,^19^. FGF-8 has been identified as a catabolic factor in rat and rabbit articular cartilage^20^. Local delivery of FGF-9 in a rat meniscal tear model of OA has been found to provide significant beneficial effect on the damaged cartilage^21^. On the other hand, results from studies investigating the therapeutic effects of FGF-2 have been conflicting. In human articular cartilage, FGF-2 plays a degenerative role in cartilage homeostasis^9^,^22^. However, in mouse joints, FGF-2 has been identified as an anabolic mediator as intra-articular injection of FGF-2 can delay cartilage degradation^23^,^24^. These discrepancy are thought due to distinctive expression patterns of FGFR1 and FGFR3 in mouse and human articular cartilage, and to the fact that FGF-2 mediates anabolic processes in mouse cartilage via its binding to FGFR3, while mediates catabolic activities via FGFR1 in human cartilage^5^.

It is reported that FGF-2 binds to FGFR1 to activate the FGFR1-Ras/PKCδ-Raf-MEK1/2-ERK1/2 signaling pathway, which leads to increased expressions of extracellular matrix degrading enzymes and down-regulated aggrecan synthesis^12^. Yan and colleagues found that blockade of Ras, PKCδ, and MAPK pathway in chondrocytes abolishes FGF-2-mediated catabolic events *in vitro* and *ex vivo*^12^. In present study, we, for the first time, provide evidence showing that a novel FGF-2/FGFR1 antagonist, G141, prevents cartilage degradation in a mouse model of OA.

Over the past three decades, several FGFR1 inhibitors, such as PD173074, SU5402 and PD166866 have been developed as candidates for the treatment of FGF signaling related diseases^11^. Most of these compounds are ATP-competitive FGFR1 inhibitors, and majority of them have failed to enter clinical application for their low-specificity and toxicity. The non-ATP-competitive inhibitors, which bind to the non-ATP binding site, possess the superior selectivity^11^. In this study, we identified a novel FGFR1 inhibitor, G141, via cell-free kinase-inhibition assay. Unlike PD173074 and other ATP-competitive FGFR1 inhibitors, G141 inhibited the activity of FGFR1 selectively in an ATP independent manner. We found that G141 had significantly stronger inhibitory effect on FGFR1 compared to that of other RTKs, such as VEGFR2, PDGFRβ, FGFR2 and FGFR3, and the inhibition of FGFR1 kinase activity by G141 did not depend on the concentration of ATP.

Next, we demonstrated that G141 inhibited the FGF-2 or IL-1β–induced upregulation of ADAMTS-5 and MMP-13 and downregulation of aggrecan and collagen type II, in human articular chondrocytes, via its inhibition on the activities of ERK1/2, JNK and p38 MAPK signaling pathway. As these 3 pathways have been found to be directly involved in the upregulation of MMP-13 in articular chondrocytes after stimulation with IL-1β or FGF-2^25^,^26^. Selective inhibition of p38 and ERK1/2 MAPK pathway has also been found to attenuate cartilage degradation in rabbit OA model and in *ex vivo* organ culture model treated with IL-1β^27^,^28^.

We further demonstrated that G141 inhibited proteoglycan degradation in FGF-2–treated human cartilage explants cultures. Most importantly, we found that the severity of cartilage degradation in a mouse model of surgically induced OA was attenuated by intra-articular injection of G141. To further investigate the cellular mechanism of the effects of G141 on articular cartilage homeostasis and OA, we examined chondrocyte hypertrophy, as chondrocyte hypertrophy can result in increased metabolic activity of articular chondrocytes and trigger unbalanced cartilage homeostasis favoring degenerative changes^29^. We found that G141 treatment reduced the expressions of type X collagen and MMP-13, most widely used markers for identifying hypertrophic chondrocytes^14^,^30^, in comparison to mice treated with vehicle after DMM surgery. These findings revealed that intra-articular injection of G141 prevented articular chondrocytes from hypertrophy in this surgical model of OA, which contributed to the inhibitory effect of G141 on OA development. The inhibitory effect of G141 on chondrocyte hypertrophy is consistent with our previous findings showing that conditional knockout of Fgfr1 in chondrocytes decreased chondrocyte hypertrophy in articular cartilage^8^.

Chondrocyte apoptosis is believed to play an important role in the pathogenesis and progression of OA^16^,^17^. Inhibition of chondrocyte apoptosis is shown to alleviate the extent of OA in a rabbit model of surgically induced OA^31^. FGF signaling pathway is highly associated with chondrocyte apoptosis^32^. Gain-of-function mutation of FGFR3 promotes chondrocyte apoptosis in thanatophoric dysplasia (TD) mice^33^. FGF-2 transgenic mice exhibit chondrodysplasic phenotype resulting from both reduced proliferation and increased apoptosis of growth plate chondrocytes^34^. FGF18 markedly reduces chondrocyte apoptosis and enhances the repair response of cartilage following cartilage insult^35^. In current study, we showed that pharmacologically inhibiting FGFR1 by G141 decreased chondrocyte apoptosis partially through its down-regulation of cleaved caspase 3 in a surgically induced mouse OA model. Our findings demonstrated that G141 positively maintains cartilage homeostasis by preventing chondrocyte apoptosis.

In conclusion, in this study, we found a novel non-ATP dependent specific FGFR1 inhibitor, G141, and for the first time, we showed that pharmacologically antagonize FGFR1 using G141 protects the knee joint cartilage from degradation in a DMM model of mouse OA, probably by suppressing the production of matrix-degrading enzymes MMP-13 and ADAMTS-5 and preventing articular chondrocytes from hypertrophy and apoptosis.

## Methods

### G141 Synthesis and kinase inhibition assays

G141 (1-methyl-4-(4-methoxyphenyl) pyrrolo (spiro-[2.2″]acenaphthene-1″-one)-spiro[3.2′]-5′-(4-methoxyphenyl)methylidenecy-clopentanones) was synthesized and identified via kinase inhibition assay as previously reported^11^,^36^. Briefly, a mixture of a bisaryl-1, 4-dien-3-one analogue (0.348 g, 1 mmol), acenaphthenequinone (0.182 g, 1 mmol), and sarcosine (0.089 g, 1 mmol) was dissolved in methanol (10 mL) and refluxed for 1 h. After completion of the reaction as evident from thin-layer chromatography (TLC), the mixture was cooled to room temperature and poured into water (50 mL). The precipitated solid was filtered and washed with water to obtain crude product, then purified by silica-gel column chromatography (petroleum ether - ethyl acetate) to give the pure product as yellow solid. The general chemical structure of G141 is shown in Fig. 1A. The kinase inhibition assay was performed using Caliper Mobility Shift Assay on EZ Reader (Caliper Life Sciences, MA) with ATP concentration at its Km value (262 μM). The compounds were tested in duplicate at 10 concentrations (5 nM–100 μM) to determine the IC_50_. In the experiments for testing the relationship between the compounds and ATP, the concentration of the substrate was constant, while the concentrations of ATP was set at 5000, 2500, 1250, 625, 313, 156, 78, and 39 μM. The global competitive inhibition fit for the compounds was performed based on percent conversion = (Vmax*X)/{km*[(1 + I/Ki)n] + X},where X is the ATP concentration, and n is the Hill coefficient.

### Isolation and culture of human articular chondrocytes

Human articular chondrocytes and cartilage explants were isolated from articular cartilage tissue harvested from patients undergoing total joint replacement surgery at Daping Hospital (Chongqing, China) because of traffic accident. Written informed consent was obtained from all subjects. Samples were collected according to protocols approved by the Institutional Review Board and Ethics Committee of Daping Hospital and the methods were carried out in accordance with the approved guidelines. Human articular chondrocytes were isolated from the cartilage according to previously described methods^8^. Isolated chondrocytes were plated into 6-well plates at a density of 1 × 10^6^ cells/well and cultured in Dulbecco’s modified Eagle’s medium/F-12 (HyClone) containing 10% fetal bovine serum (Gibco) and 50 units/ml of penicillin and streptomycin (HyClone). At 90% confluence, human chondrocytes were cultured under serum-free conditions for 24 hours. The chondrocytes were incubated with G141 (5 μM and 10 μM) for 1 hour before treatment with 20 ng/ml FGF-2 (Peprotech) or 20 ng/ml IL-1β (Peprotech) for 24 hours.

### Human articular cartilage explants culture

For cartilage explants culture, full thickness femur head cartilage tissue was cut into pieces of ~2 mm–3 mm. Following 48 hours of culture in Dulbecco’s modified Eagle’s medium/F-12 containing 10% fetal bovine serum and 50 units/ml of penicillin and streptomycin, explants were treated with FGF-2 (50 ng/ml) and G141 (5 μM and 10 μM) for 14 days under serum-free conditions (with ITS)^24^. Following 14 days culture, the medium was collected and the explants were fixed in 4% paraformaldehyde.

### Dimethylmethylene blue assay

The DMMB dye binding assay was performed to analyze glycosaminoglycan (GAG) release of the cultured explants as previously described^13^,^37^. Briefly, 250 μl of DMMB reagent was added to 40 μl of culture medium and the absorbance was measured at 525 nm. Using different concentrations of chondroitin sulfate (Sigma-Aldrich) to plot a standard curve and then the amount of GAG released was determined by this standard curve. GAG released into the medium was normalized as mass of GAG per milliliter (ml) of culture medium.

### Western blotting

Human articular chondrocyte cultures were extracted using RIPA lysis buffer containing protease inhibitors (Roche). Protein was extracted from mouse whole joints following removal of the skin and muscle bulk. Tissue was snap-frozen and then extracted using RIPA lysis buffer. Equal amount of protein samples (30 μg) were dissolved by 12% sodium dodecyl sulfate–polyacrylamide electrophoresis gels and transferred onto a polyvinylidene difluoride membrane. After being blocked with 5% nonfat milk in Tris buffered saline–Tween buffer, the membrane was probed with primary antibodies specific for phosphorylated and total ERK1/2 (CST), p38 (CST), JNK (CST), MMP-13 (Millipore), ADAMTS-5 (Abcam), and phosphorylated and total FGFR1 (Santa) followed by secondary antibodies. The signal was detected using chemiluminescent (Pierce) according to the manufacturer’s instruction. The antibody specific for β-actin (Sigma) was applied to normalize the protein expression levels.

### Real-time qPCR

Total RNA was isolated from human chondrocyte cultures using the Trizol reagent (Invitrogen). Total RNA was extracted from mouse whole joints following removal of the skin and muscle bulk. Tissue was snap-frozen and then extracted using Trizol reagent as previously described^23^. Real-time qPCR was performed using Max3000 PCR machine (Stratagene) and SYBR Premix Ex TaqTM kit (Takara). The sequences for the primers used were as follows: human GAPDH, 5′-CGCTCTCTGCTCCTCCTGTT-3′ (forward) and 5′-CCATGGTGTCTGAGCGATGT-3′ (reverse); human MMP-13, 5′-AAGG-AGCATGGCGACTTCT-3′ (forward) and 5′-TGGCCCAGGAGGAAAAGC-3′ (reverse); human ADAMTS-5, 5′-TGGCTCACGAAATCGGACA-3′ (forward) and 5′-GGAACCAA-AGGTCTCTTCACAGA-3′ (reverse); human aggrecan, 5′-AGGCAGCGTGATCCTTACC3′ (forward) and 5′-GGCCTCTCCAGTCTCATTCTC-3′ (reverse); human Collagen type II, 5′-CGTCCAGATGACCTTCCTACG-3′ (forward) and 5′-TGAGCAGGGCCTTCTTGAG-3′ (reverse); mouse FGF-2, 5′-GCGACCCACACGTCAAACTA-3′ (forward) and 5′-CCGTC-CATCTTCCTTCATAGC-3′ (reverse); mouse IL-1β, 5′-GAAATGCCACCTTTTGACAGT-G-3′ (forward) and 5′-TGGATGCTCTCATCAGGACAG-3′ (reverse); mouse Cyclophili A, 5′-CGAGCTCTGAGCACTGGAGA-3′ (forward) and 5′-TGGCGTGTAAAGTCACCACC-3′ (reverse). All samples were measured in triplicate, and the GAPDH and Cyclophili A were amplified as an internal control.

### Mouse Surgically induced model of OA

Animal experiments were performed according to protocols approved by the Laboratory Animal Welfare and Ethics Committee of the Third Military Medical University (Chongqing, China) and the methods were carried out in accordance with the approved guidelines. Destabilization of the medial meniscus (DMM) surgery was made on the right knee joints of 10-week-old male C57BL/6 mice, as previously described^38^. Sham surgery were performed with medial capsulotomy only. All mice were then allowed to move freely and take food and water ad libitum after surgery.

### Intra-articular injection of G141

DMM mice received intra-articular injection of 10 μl of 10 μM G141 in PBS twice a week for 4 and 8 weeks immediately after DMM surgery. The control group received intra-articular injection of 10 μl of PBS only. At 4 and 8 weeks post DMM, animals were sacrificed and the knee joints were harvested and fixed in 4% paraformaldehyde.

### Histology

Knee joints of the mice were decalcified in 20% formic acid, and embedded in paraffin. 5-mm thick sections were cut sagittally through the medial knee joints and stained with stained Safranin O- fast green to assess cartilage destruction as previously described^8^. The Osteoarthritis Research Society International (OARSI) recommended subjective scoring system was used to histologically grade the severity of the cartilage destruction^39^. The score for an individual joint was expressed as a summed score for the medial femora and medial tibiae within each joint separately.

### Immunohistochemistry

Immunohistochemistry was performed on sagittal sections of paraffin-embedded knee joints. After being deparaffinized using xylene and deprived of endogenous peroxidase activity with 3% H_2_O_2_, and antigen retrieval with 0.1% trypsin, sections were incubated with rabbit anti–MMP-13 polyclonal antibody (1:200 dilution; Abcam), rabbit anti–type X collagen polyclonal antibody (1:200 dilution; Millipore), rabbit anti- cleaved caspase-3 polyclonal antibody (1:100; Boster) overnight at 4 °C. After warming and cleaning, sections were incubated with horseradish peroxidase–conjugated secondary antibodies for 30 min at 37 °C. Finally, sections were stained with diaminobenzidine (DAB) kit and counterstained with methyl green.

### TUNEL staining

*In situ* cell death detection kit (Roche) was used to detect apoptotic articular cartilage chondrocytes according to the manufacturer’s instruction.

### Statistical analysis

The numeric data were expressed as the mean ± SD. Differences between 2 groups were evaluated using Student’s t-test. Analysis of variance (ANOVA) was used for comparisons of 3 or more groups followed by Tukey post hoc test (SPSS program version 13.0). P < 0.05 were considered statistically significant.

## Additional Information

**How to cite this article**: Xu, W. *et al.* A novel fibroblast growth factor receptor 1 inhibitor protects against cartilage degradation in a murine model of osteoarthritis. *Sci. Rep.* **6**, 24042; doi: 10.1038/srep24042 (2016).

## Supplementary Material

Supplementary Information
