# Supplementary material for: A novel fibroblast growth factor receptor 1 inhibitor protects against cartilage degradation in a murine model of osteoarthritis
Source: Sci Rep. 2016 Apr 4;6:24042. doi: 10.1038/srep24042 (PMC4819196; doi:10.1038/srep24042)
Supplement: Supplementary Information [file srep24042-s1.pdf]

A novel fibroblast growth factor receptor 1 inhibitor protects against cartilage degradation in a murine model of osteoarthritis

Wei Xu[1], Yangli Xie[1], Quan Wang[1], Xiaofeng Wang[1], Fengtao Luo[1], Siru Zhou[1], Zuqiang Wang[1], Junlan Huang[1], Qiaoyan Tan[1], Min Jin[1], Huabing Qi[1], Junzhou Tang[1], Liang Chen[1], Xiaolan Du[1], Chengguang Zhao[2], Guang Liang[2*] and Lin Chen[1*]

[1]Department of Rehabilitation Medicine, Center of Bone Metabolism and Repair, State Key Laboratory of Trauma, Burns and Combined Injury, Trauma Center, Research Institute of Surgery, Daping Hospital, Third Military Medical University, Chongqing 400042, China

[2]Institute of Biological and Natural Medicine, School of Pharmaceutical Science, Wenzhou Medical University, Wenzhou 325035, China

[*]Corresponding authors**:** Lin Chen (linchen70@163.com) and Guang Liang (wzmcliangguang@163.com).

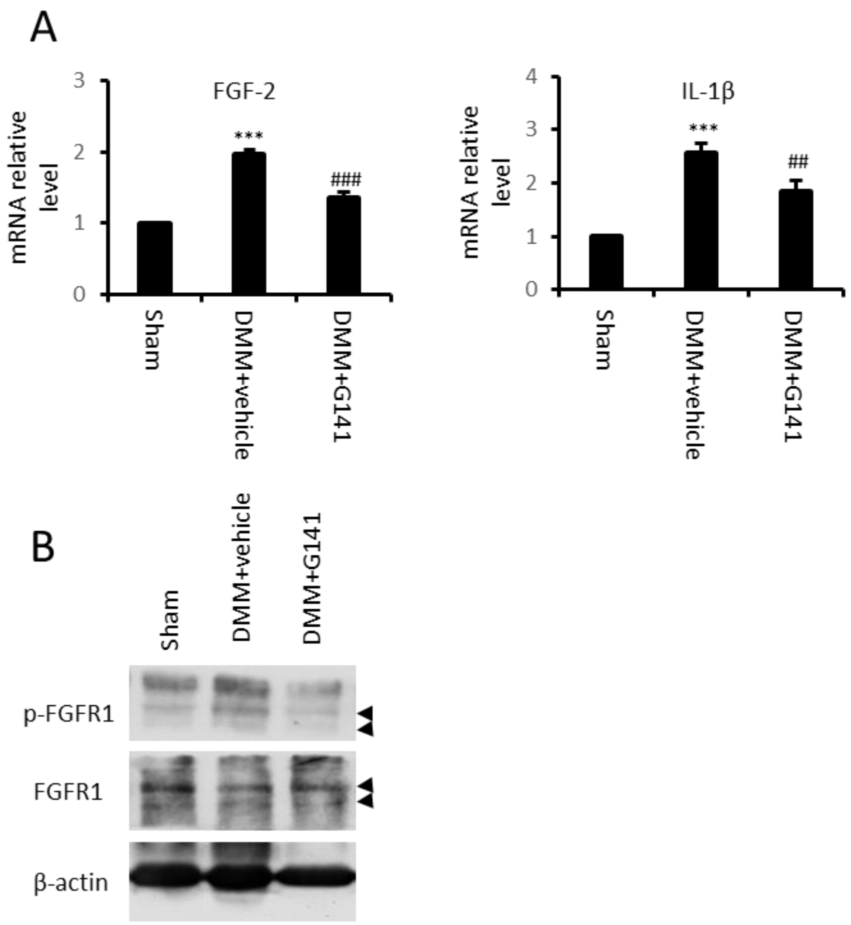

Figure S1

FGF-2 and IL-1β mRNAs are up-regulated and FGFR1 activity is enhanced in mouse knee joints after DMM surgery. 10-week-old male C57BL/6 mice were subjected to sham operation or DMM surgery followed by intra-articular injection of 10 μM G141 or PBS twice a week for 2weeks, immediately after DMM surgery. A, total RNA was isolated from the knee joints, and levels of mRNA of the FGF-2 and IL-1β were detected by Real-time qPCR (Values are the mean ± SEM. n=3, ***$p<0.001$ versus sham-operated knee joints, ##$p<0.01$ versus DMM-operated knee joints, ###$p<0.001$ versus DMM-operated knee joints). B, total protein was extracted from knee joints and analyzed by Western blotting using antibodies specific for phosphorylated or total FGFR1.
